# Pluripotent Stem Cells for Brain Repair: Protocols and Preclinical Applications in Cortical and Hippocampal Pathologies

**DOI:** 10.3389/fnins.2019.00684

**Published:** 2019-08-06

**Authors:** Claudia Alia, Marco Terrigno, Irene Busti, Federico Cremisi, Matteo Caleo

**Affiliations:** ^1^CNR Neuroscience Institute, National Research Council (CNR), Pisa, Italy; ^2^Laboratory of Biology, Scuola Normale Superiore, Pisa, Italy; ^3^Department of Neuroscience, Psychology, Drugs and Child Health Area, School of Psychology, University of Florence, Florence, Italy; ^4^Biophysics Institute (IBF), National Research Council (CNR), Pisa, Italy; ^5^Department of Biomedical Sciences, University of Padua, Padua, Italy; ^6^Padua Neuroscience Center, University of Padua, Padua, Italy

**Keywords:** pluripotent stem cells, stroke, cortex, hippocampus, brain injuries, brain repair, cell-based therapy, differentiation protocols

## Abstract

Brain injuries causing chronic sensory or motor deficit, such as stroke, are among the leading causes of disability worldwide, according to the World Health Organization; furthermore, they carry heavy social and economic burdens due to decreased quality of life and need of assistance. Given the limited effectiveness of rehabilitation, novel therapeutic strategies are required to enhance functional recovery. Since cell-based approaches have emerged as an intriguing and promising strategy to promote brain repair, many efforts have been made to study the functional integration of neurons derived from pluripotent stem cells (PSCs), or fetal neurons, after grafting into the damaged host tissue. PSCs hold great promises for their clinical applications, such as cellular replacement of damaged neural tissues with autologous neurons. They also offer the possibility to create *in vitro* models to assess the efficacy of drugs and therapies. Notwithstanding these potential applications, PSC-derived transplanted neurons have to match the precise sub-type, positional and functional identity of the lesioned neural tissue. Thus, the requirement of highly specific and efficient differentiation protocols of PSCs in neurons with appropriate neural identity constitutes the main challenge limiting the clinical use of stem cells in the near future. In this Review, we discuss the recent advances in the derivation of telencephalic (cortical and hippocampal) neurons from PSCs, assessing specificity and efficiency of the differentiation protocols, with particular emphasis on the genetic and molecular characterization of PSC-derived neurons. Second, we address the remaining challenges for cellular replacement therapies in cortical brain injuries, focusing on electrophysiological properties, functional integration and therapeutic effects of the transplanted neurons.

## Introduction

Brain injuries represent a large variety of disabling pathologies. They may originate from different causes and affect distinct brain locations, leading to an enormous multiplicity of various symptoms ranging from cognitive deficits to sensorimotor disabilities. They can also result in secondary disturbances, such as epileptic foci, which occur within the lesioned and perilesional tissues (Herman, 2002). Indeed, frequently a secondary functional damage can take place in a region distant from the first insult (e.g., the hippocampus after traumatic brain injury), providing an explanation for cognitive and memory deficits arising after a brain lesion (Girgis et al., 2016). Brain injuries can have traumatic or non-traumatic etiologies, including focal brain lesions, anoxia, tumors, aneurysms, vascular malformations, encephalitis, meningitis and stroke (Teasell et al., 2007). In particular, stroke covers a vast majority of acquired brain lesions. *Stroke* is usually referred to as a sudden cerebrovascular dysfunction leading to focal deficits and/or impairment of global brain functions and lasting more than 24 h (Mackay and Mensah, 2004). Since brain function is strictly dependent on a constant supply of oxygen and glucose, normally assured by blood circulation, a sudden block of blood supply determines suppression of neural function in less than 1 min, primarily due to interference with synaptic functions (Hofmeijer and Van Putten, 2012). Brief blood deprivations may cause only a reversible damage, which becomes permanent only if the circulation is not promptly restored (Krnjević, 2008; Vrselja et al., 2019). The neocortex represents the highest level of cognitive and sensorimotor integration, and it is therefore not surprising that, independently of different etiologies, lesions occurring in the cerebral cortex are particularly impacting on the clinical phenotype (Delavaran et al., 2013). For example, an insult occurring in the motor cortex results in functional impairment of one or more body parts contralateral to the infarct. The degree of the motor impairment depends on many factors, such as the extent of the lesion, the identity of the damaged region and the effectiveness of the initial neuroprotective interventions.

Following stroke, there is a window of neuroplasticity during which the greatest gains in recovery occur (Zeiler and Krakauer, 2013). Indeed, in the first weeks after stroke a limited spontaneous restoration of function may be observed, and about 30% of stroke survivors are able to carry on everyday activities (Activity of Daily Living or ADLs, i.e., eating, drinking, walking, etc.) without any help (Mozaffarian et al., 2014). However, other patients do not recover at all (Winters et al., 2018). In particular, impairments of upper and lower limbs make very hard to retain a sufficient degree of independence in ADLs. These impairments can be ameliorated with a variable degree of success, through rehabilitation of the affected body parts, including several physical activities improving strength and coordination of the affected muscles and promoting recover of motility. Furthermore, combining rehabilitation with treatments that enhance neuroplasticity has been demonstrated to boost recovery (Alia et al., 2017; Spalletti et al., 2017) but further steps forward in the field are necessary for clinical translation.

Besides physical rehabilitation and plasticizing treatments, another therapeutic approach is cell-based therapy, which has been pioneered in the therapy of Parkinson Disease (PD). Indeed, initial studies showed that fetal dopaminergic neurons grafted in the striatum ameliorated PD symptoms, both in animal models (Herman and Abrous, 1994) and in patients (Lindvall et al., 1990; Kordower et al., 1998). Since fetal transplantation poises both ethical issues and technical challenges (Robertson, 2001), other non-neural cells, such as mesenchymal stem cells (MSCs), may represent a more accessible alternative. In fact, MSCs can be readily derived from various sources, show a low immunogenic effect and proved to be beneficial in stroke treatment (Eckert et al., 2013; Zhang and Chopp, 2013). Notably, MSCs-conditioned medium alone is sufficient for a similar therapeutic effect, suggesting that the beneficial effect is likely due to a bystander effect and trophic support, rather than actual cell replacement (Eckert et al., 2013). Recent clinical trials proved the safety of immortalized neural cells when stereotaxically injected in the brain of patients with stable paresis of the arm following an ischemic stroke (Kalladka et al., 2016). Finally, induced pluripotent stem cells (iPSCs) are easily accessible, since they can be reprogrammed starting from somatic cells, can be *in vitro* differentiated in the desired type of neurons and, being autologously derived and carry low risk of immune rejection (Jung et al., 2012; Trounson and DeWitt, 2016).

Due to the high clinical relevance of cerebral lesions, protocols to differentiate neural stem cells in telencephalic neurons are highly valuable. These protocols are frequently inspired by findings in neural development.

## Developmentally Inspired Stem Cells Differentiation Paradigms

Attempts to generate identity-specific cortical neurons require exploitation of the right embryonic developmental signals in a culture dish. The six layers of the mammalian neocortex originate from inside-out in a timely regulated manner during embryonic development and are characterized by a specific pattern of gene expression and connectivity (reviewed in: Rubenstein, 2011; Bronner and Hatten, 2013; Greig et al., 2013; Lodato and Arlotta, 2015). Both intrinsic and environmental factors control the progressive restriction of competence of cortical progenitors resulting in the sequential generation of the different cortical neurons, with deeper layer cortical neurons generated earlier than upper layer neurons (Gorski et al., 2002; Kriegstein and Noctor, 2004; Molyneaux et al., 2007).

**FIGURE 1:**
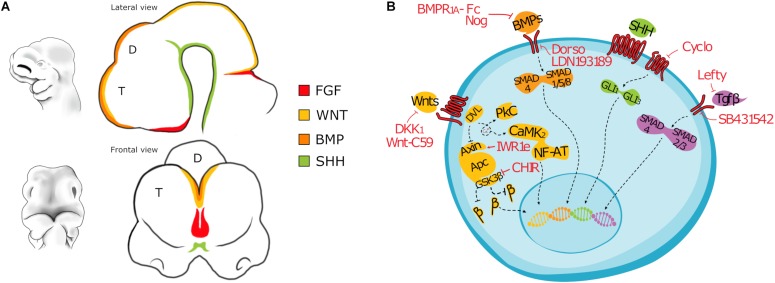
**(A)** Drawing depicting the main morphogens involved in the patterning of the forebrain of a mouse embryo (E10). T, Telencephalon; D, Diencephalon. **(B)** Cartoon showing main signaling pathways active in differentiating PSC and the inhibitors most commonly used. BMP signaling: Noggin, Bone Morphogenetic Protein Receptor 1A Fc chimera (BMPR1A-Fc), LDN193189, or Dorsomorphin; WNT signaling: CHIR99021, Dickkopf-related protein 1 (Dkk1), IWR-1-endo, 53AH or WNT-C59; TGFβ signaling: Lefty, SB431542; SHH signaling: Cyclopamine. β: β-catenin (CTNNB1).

The hippocampus arises from the invagination of the dorsal midline of the telencephalon, adjacent to the cortical hem. The boundaries between choroid plexus, cortical hem and hippocampus are defined early in development by the non-overlapping expression of molecular markers signatures. In particular, BMP4/7 are strongly expressed by the choroid plexus (Grove et al., 1998; Bulchand et al., 2001), while the cortical hem expresses high level of WNT3a, WNT2, and WNT5, whose signaling is essential for the correct development of the hippocampus (Grove et al., 1998) (Figure 1A). Consistently, experiments in which the cortical hem is ablated, or hem-dependent WNT expression is abrogated or compromised, showed the crucial role of the cortical hem in the hippocampal development (Grove et al., 1998; Grove and Tole, 1999; Lee et al., 2000). The Dentate Gyrus (DG), the CA1 and CA3 layers are the different fields forming the hippocampal formation; each one has its own cellular composition, morphology, connectivity and expresses specific molecular markers. In particular, KA1, a glutamate receptor subunit, is detectable in the CA3, while SCIP, a POU-domain transcription factor, is expressed in the CA1. The pyramidal neurons of the CA1-3 originate in the more dorsal ventricular zone of the hippocampus and then migrate toward the pial surface on a glial scaffold (Nadarajah et al., 2001), while the DG cells arise from a smaller and specialized medial region close to the fimbria, the dentate neuroepithelium (Danglot et al., 2006). Furthermore, new neurons are generated in the hippocampus of rodents throughout life, while cortical neurogenesis rapidly subsides after birth (Eriksson et al., 1998; van Praag et al., 2002). This makes hippocampus the region of choice to assay the ability of *in vitro* produced PSC-derived hippocampal-like neural cells to make projections and to send them to appropriate targets.

Pluripotent stem cells cultured in low-density, serum-free and feeder-free conditions, in absence of any exogenous growth factor, readily differentiate into neural progenitor cells (Tropepe et al., 2001; Ying et al., 2003). This process faithfully reproduces *in vivo* neurogenesis (reviewed in: van den Ameele et al., 2014; Brown et al., 2018; Varrault et al., 2019), following the same timely regulated sequential neurogenic waves observed in vivo (Eiraku et al., 2008; Gaspard et al., 2008; Bertacchi et al., 2013; Molyneaux et al., 2015), which is therefore conserved *in vitro* (Shen et al., 2006). In fact, differentiating PSCs give rise to neural rosettes expressing the early neural markers Sox1, Pax6 and Nestin and closely resembling the early embryonic neural tube (Perrier et al., 2004). When dissociated and replated, PSC-derived neural rosettes give rise to neural progenitor cells (NPCs) which subsequently differentiate into a heterogeneous mixture of different subtypes of neurons (GABAergic, dopaminergic, serotonergic, and cholinergic), astrocytes and oligodendrocytes (Zhang et al., 2001; Barberi et al., 2003). Furthermore, PSCs cultured in this conditions spontaneously acquire an anterior dorsal neural identity (Watanabe et al., 2005; Pankratz et al., 2007), while the addition of growth factors to the medium can steer the differentiation of PSCs toward midbrain/hindbrain (Kawasaki et al., 2000; Perrier et al., 2004) or spinal cord (Wichterle et al., 2002; Li et al., 2009) fates. Thus, PSCs are first committed toward an anterior neural fate and successively patterned by caudalizing factors to acquire a more posterior neural identity, supporting a mammalian model of default neural induction (Muñoz-Sanjuán and Brivanlou, 2002; Smukler et al., 2006; Stern, 2006; Levine and Brivanlou, 2007; Gaulden and Reiter, 2008). Finally, recent evidence suggest that the regionalization of the presumptive neural ectoderm precedes neural commitment; indeed these results further support the notion that FGF, WNT and RA signaling are required for the acquisition of posterior neural identity by PSCs differentiating *in vitro* (Metzis et al., 2018).

The positional identity of neural cells obtained by pluripotent cell during *in vitro* differentiation is often established by the simple study of their neurotransmitter phenotype (Eiraku and Sasai, 2011; Shi et al., 2012; Yu et al., 2014; Shiraishi et al., 2017), or by deeper investigation of their molecular nature through methods of global gene expression analysis (Bertacchi et al., 2013, 2014; Espuny-Camacho et al., 2013; Leemput et al., 2014; Edri et al., 2015; Yao et al., 2017; Terrigno et al., 2018a). However, specific neural subtypes present in different areas of the adult brain show, in addition to distinct gene expression profiles, also defined connectivity patterns, electrophysiological proprieties and morphologies (Cadwell et al., 2015; Fuzik et al., 2015; Tiklová et al., 2019). Whereas the molecular signature of neurons can be established prior their transplantation, the evaluation of all the other parameters require *in vivo* analysis. Understanding how to obtain a precise subtype of neuron, patterned with the proper temporal and positional identity, is pivotal and might have considerable repercussion on possible clinical applications.

**FIGURE 2:**
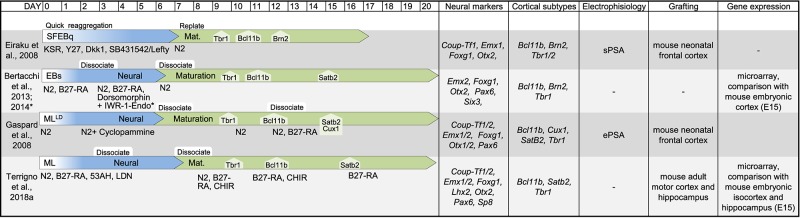
Table showing original reports of differentiation protocols of murine PSCs in cortical and hippocampal neurons. For each entry we indicated (from left to right): reference, schematic layout of the protocol, neural markers, cell-type markers, electrophysiological proprieties, target of transplantation assay to assess integration, global gene expression analysis to identify cell positional identity. If the information was available, we indicated the time of emergence of main cortical cell types (TBR1, BCL11B, BRN2, SATB2, and CUX1). Due to the sheer number of studies on this topic, derivative reports of minor modifications to previously published protocols are not included in this table. SFEBq, serum-free culture of embryoid body-like aggregates; EB, embryoid body; ML, monolayer; LD, low-density; sPSA, spontaneous post-synaptic activity; ePSA, evoked post-synaptic activity.

**FIGURE 3:**
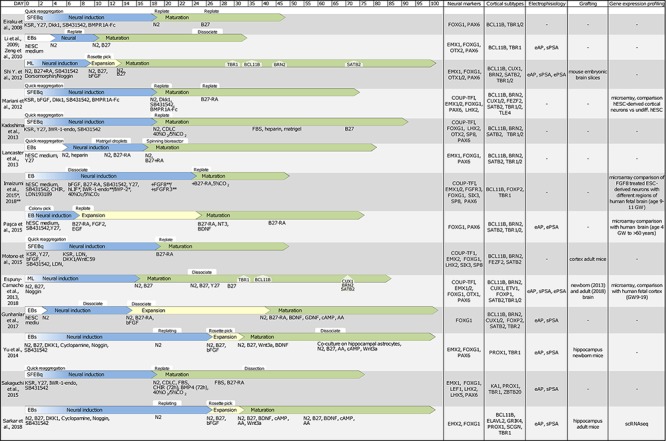
Table showing original reports of differentiation protocols of human PSCs in cortical and hippocampal neurons. With same criteria as those indicated in Figure 2 SFEBq, serum-free culture of embryoid body-like aggregates; EB, embryoid body; ML, monolayer; scRNAseq, single cell RNA sequencing; eAP, evoked action potential; sPSA, spontaneous post-synaptic activity; ePSA, evoked post-synaptic activity.

In this review, we have considered available protocols to differentiate murine and human pluripotent stem cells into cortical- or hippocampal-like stem cells (Figures 2, 3) expressing at least a forebrain marker (FOXG1 or EMX1/2) and an early cortical or hippocampal marker (TBR1/2, BCL11B or PROX1).

### Derivation of Cortical-Like Neurons From Pluripotent Stem Cells

The derivation of cortical-like neurons from PSCs was first reported a decade ago by the laboratories of Pierre Vanderhaeghen (Gaspard et al., 2008) and Yoshiki Sasai (Eiraku et al., 2008). Interestingly, Sasai’s protocol is also the first report on 3D cortical organoid from both murine and human PSCs obtained by inhibiting both the endogenous WNT and TGFβ signaling. On the contrary, in Vanderhaeghen’s protocol PSCs are cultured as low density adherent monolayer in a minimal, chemically defined medium supplied with a SHH inhibitor, Cyclopamine. Indeed, these authors pioneered the generation of cortical-like cells from PSCs, paving the road for the subsequent studies on human PSCs and induced PSCs (Figures 2, 3). Notably, cortical-like cultures, expressing anterior neural markers (FOXG1 and EMX1/2) and enriched in several cortical cell types (BCL11B, TBR1/2, SATB2, BRN2, and CUX1), can be obtained by simply differentiating PSCs, cultured in suspension as embryoid bodies (Ebs), or as attached monolayer, in a chemically defined minimum medium, devoid of any grow factors (Gaspard et al., 2008; Li et al., 2009; Zeng et al., 2010; Lancaster et al., 2013). However, most authors block endogenous posteriorizing or ventralizing factors (Figure 1) to promote the commitment toward an anterior dorsal fate and enrich the culture in cortical cell types (Eiraku et al., 2008; Gaspard et al., 2008; Mariani et al., 2012; Shi et al., 2012; Bertacchi et al., 2013, 2014; Espuny-Camacho et al., 2013, 2018; Kadoshima et al., 2013; Renner et al., 2013; Yu et al., 2014; Paşca et al., 2015; Sakaguchi et al., 2015; Motono, 2016; Gunhanlar et al., 2017; Sarkar et al., 2018; Terrigno et al., 2018a), including BMP inhibitors (Noggin, BMPR1A-Fc, LDN193189, or Dorsomorphin), WNT inhibitors (Dkk1, IWR-1-endo, 53AH, or WNTC59) and TGFβ inhibitors (Lefty, SB431542). In addition, the default fate of PSC-derived neural progenitors appears to be dorsal, thus fewer protocols include the inhibition of SHH signaling (Gaspard et al., 2008; Yu et al., 2014; Sarkar et al., 2018).

Interestingly, by default PSC-derived cortical neurons appear to acquire mainly limbic and occipital cortical identities (Gaspard et al., 2008; Bertacchi et al., 2013; Terrigno et al., 2018b). In fact, cortical-like cultures show high levels of posterior cortical markers, namely COUP-TF1 (Gaspard et al., 2008; Bertacchi et al., 2013; Terrigno et al., 2018b), an orphan nuclear receptor that is expressed in a high posterior-lateral to low anterior-medial expression gradient by both progenitors and cortical neurons, and is required to balance the antero-posterior patterning of the neocortex (Armentano et al., 2007; Liu et al., 2010). In addition, heterotopic transplantation experiments into the adult murine cortex showed patterns of projection strikingly similar to grafted embryonic visual cortical cells (Michelsen et al., 2015; Espuny-Camacho et al., 2018), supporting the hypothesis of a default occipital fate for PSC-derived neurons. Indeed, treatment with the morphogen FGF8 can induce frontal motor cortical fates in PSC-derived cultures (Eiraku et al., 2008; Kadoshima et al., 2013; Imaizumi et al., 2018; Terrigno et al., 2018b). Intriguingly, Motono et al. reported the generation of PSC-derived anterior cortical neurons (low COUP-TF1 / high SP8 expression) by blocking both the canonical and non-canonical WNT signaling with an inhibitor of Porcupine (PORCN), a membrane-bound O-acyltransferase involved in the palmitoylation, and subsequent secretion, of WNT proteins (Motono et al., 2016). Although these results remain to be confirmed by whole genome expression profiling of the cells and a more extensive analysis of their morphology and connectivity following homotopic and heterotopic transplantation, they are yet another example of how culture conditions profoundly influence the positional identity of PSC-derived cortical neurons. Finally, since different protocol to obtain cells with anterior cortical identity are now available (Eiraku et al., 2008; Kadoshima et al., 2013; Motono, 2016; Imaizumi et al., 2018; Terrigno et al., 2018b), further studies should focus on PSC-derived neurons with motor cortical identity transplanted in the damaged motor cortex.

Besides positional identity, the cellular composition of the cell culture has great clinical implications, since the projection pattern of transplanted PSC-derived neurons varies according to their cellular identity. That is, TBR1- and BCL11B-positive PSC-derived neurons transplanted in the adult brain generate, respectively, cortico-thalamic and corticofugal projections (Espuny-Camacho et al., 2013; Michelsen et al., 2015), consistently with the connectivity of normal TBR1- and BCL11B-positive cells. In fact, differentiating PSCs can generate all the cell-types of the six cortical layers (van den Ameele et al., 2014), although culture conditions strongly influence their ratio. Indeed, the production of cortical pyramidal neurons by PSCs cultured in minimal medium as monolayer cultures is strongly skewed toward lower layer identity (Gaspard et al., 2008; Espuny-Camacho et al., 2013). However, the proportion of upper cortical neurons can be increased by supplying retinoids during dual SMAD inhibition (Shi et al., 2012), by transplantation in host brain (Espuny-Camacho et al., 2013) or by culturing PSCs as aggregates (Eiraku et al., 2008; Mariani et al., 2012; Lancaster et al., 2013). Organoid cortical cultures more faithfully reproduce several features of *in vivo* corticogenesis (reviewed in Lancaster and Knoblich, 2014; Di Lullo and Kriegstein, 2017; Giandomenico and Lancaster, 2017), such as migration of newborn neurons on radial glia processes (Bamba et al., 2017), interkinetic nuclear migration (Paşca et al., 2015), and interactions and connections between different regions of the neuroepithelium, such as the interneuron migration and cortical invasion in 3D co-cultures of dorsalized and ventralized PSC-derived neurons (Birey et al., 2017). Finally, although these methods do not allow the identification of the signals responsible for PSC specification, aggregate cultures display higher axonal outgrowth (Chandrasekaran et al., 2017) and cell survival (Daviaud et al., 2018) following cortical transplantation, holding great promises for *in vitro* modeling of neurodegenerative diseases (Quadrato et al., 2016).

During telencephalic development, GABAergic interneurons are generated in the subpallial caudal and medial ganglionic eminence (CGE and MGE) and migrate tangentially to the cortex and hippocampus (Marín and Rubenstein, 2001; Xu et al., 2004; Hansen et al., 2013; Ma et al., 2013). Several neurological disorders are caused by dysfunctions of the GABAergic system and in the last decades GABAergic cell-based therapies have emerged as a promising treatment in restoring the lost balance between inhibitory and excitatory circuits (Danjo et al., 2011; reviewed in Alvarez Dolado and Broccoli, 2011; Hunt et al., 2013; Shetty and Upadhya, 2016). Furthermore, several groups were able to efficiently generate forebrain GABAergic interneurons from PSCs (Gaspard et al., 2008; Li et al., 2009; Zeng et al., 2010; Falk et al., 2012; Germain et al., 2013; Liu et al., 2013; Maroof et al., 2013; Nicholas et al., 2013; Tornero et al., 2013; Yuan et al., 2015; Liao et al., 2016; Sun et al., 2016; Xu et al., 2016). However, due to the ventral origin of the forebrain interneurons, PSCs committed in culture to dorsal telencephalic fates, such as cortex and hippocampus, generate mainly glutamatergic neurons (Zhang et al., 2001; Bibel et al., 2004; Eiraku et al., 2008; Gaspard et al., 2008, 2009; Bertacchi et al., 2013, 2014; Espuny-Camacho et al., 2013; Yu et al., 2014; Michelsen et al., 2015; Livesey et al., 2016; Motono, 2016; Sarkar et al., 2018; Terrigno et al., 2018a). Since GABA-ergic inhibition is essential for maintaining the balanced activity of cortical neural circuits and for the development of neural network activity (Ben-Ari et al., 2007), grafting of PSC-derived cortical or hippocampal cultures enriched with interneurons could have significant clinical repercussions and should be further investigated.

### Derivation of Hippocampal-Like Neurons From Pluripotent Stem Cells

The first report of PSC-derived neural progenitors committed to a medial pallial identity was by Sasai and colleagues (Eiraku et al., 2008). Since then, several authors described methods to commit PSC to either choroid plexus or hippocampus (Figures 1, 2; Yu et al., 2014; Sakaguchi et al., 2015; Sarkar et al., 2018; Terrigno et al., 2018a). Most of these protocols share an initial phase of WNT, BMP and TGFβ inhibition, followed by activation of BMP and WNT signaling, by either suppling WNT3a or a small-molecule inhibitor of GSK3 (CHIR). In particular, Sakaguchi et al. showed that a short-term activation of WNT and BMP signaling in anteriorized neural progenitors specifies a hippocampal primordium-like tissue *in vitro*, expressing the hippocampal markers ZBTB20, KA1 and PROX1; while a prolonged treatment results in increased expression of LMX1A and TTR, markers of choroid plexus (Sakaguchi et al., 2015). Consistently, other authors showed that double inhibition of WNT and BMP endogenous signaling in differentiating PSC followed by timely activation of WNT signaling results in hippocampal specification (Yu et al., 2014; Sarkar et al., 2018; Terrigno et al., 2018a). In particular, the laboratory of Fred Gage showed that a prolonged treatment with WNT3a results in the commitment of PSC to DG fate, while a shorter treatment and with a lower concentration of WNT3a specifies CA3 identity (Yu et al., 2014; Sarkar et al., 2018). Notably, PSC-derived hippocampal-like neurons, but not cortical-like cells, transplanted into adult hippocampus were capable of long-term survival, contacted relevant targets of the hippocampal formation and formed functional synaptic contacts with the host network, further confirming the importance of the identity match between transplanted cells and surrounding environment (Terrigno et al., 2018a).

## *In Vivo* Integration and Therapeutic Effect of Grafted Exogenous Neural Cells in the Brain

Once obtained the cell type(s) of interest, the next step is to evaluate if these cells can properly integrate in the host tissue, sending short and long projections to their natural targets and rebuilding damaged circuitry. Neurons suitable for transplantation must be able to integrate into the host context, produce the appropriate type of neurotransmitter and neurotransmitter receptors and develop synapses with functionally relevant targets and electrophysiological properties of mature neurons.

### Anatomical Characterization of Grafted Neurons in the Host Circuits

Initial reports showed that the outgrowth from grafts of fetal neurons in adult host brain was limited and dependent on the site of injection (Gonzalez et al., 1988; Isacson et al., 1988; Fricker et al., 1999). The first consistent observation of long-distance axons and extensive innervation of the host brain by fetal transplants following experimental deletion of cortical projection neurons was reported by Hernit-Grant and Macklis (1996) and Fricker-Gates et al. (2002). However, in these studies the specificity of efferent projections from the grafted cells toward appropriate host target sites was not assessed. Subsequently, Gaillard et al., 2007 described the reconstruction of damaged cortical circuitry in adult mice and the formation of long-range projections targeting the appropriate host regions, notably the pyramidal tract and the spinal cord. 2 months after the transplantation of fetal cortical tissue in the damaged motor cortex, they observed outgoing projections toward most of the cortical and subcortical targets of the cortical motor neurons, comparable to those established by neurons of the contralesional motor cortex. Furthermore, they verified that most of the grafted neurons sent projections along motor pathways, by injecting the retrograde tracer cholera toxin in the brain regions where fibers were found (i.e., thalamus and dorsolateral caudate putamen). Interestingly, the fact that in the same study visual grafts were not competent to replace degenerating motor pathways suggests that transplanted cells retain positional identity and functional capability of the donor. Altogether, these initial reports showed that transplanted embryonic neurons are able to extend long-range projections to distant targets with great specificity, suggesting that guidance cues persist in the adult brain or are re-expressed after injury, although no electrophysiological and/or behavioral evidence was provided to corroborate the anatomical observations (Gaillard et al., 2007).

More recently, Falkner et al. (2016) performed *in vivo* 2-photon imaging of embryonic cortical neurons transplanted 7 days after ablation of layer II-III callosal projecting neurons in the monocular visual cortex, evaluating longitudinal structural maturation, morphology, dendrite elongation and spine refinement of grafted neurons. Notably, already at 3–4 days post-transplantation, grafted neurons extended branched neurites, with identifiable growth cone and boutons, followed by the formation of apical dendrites which reached complete development within 4 weeks post-transplantation (wpt). Spine and bouton density greatly increased up to 4 wpt and then reached a plateau, while turnover rates increased at 5 wpt and subsequently stabilized after 8 wpt. Interestingly, as soon as 5 wpt, grafted cells displayed appropriate identity of pyramidal neurons and projected to the natural targets of V1 neurons, including ipsilateral visual areas, retrosplenial and entorhinal cortices and the corpus callosum. Furthermore, grafted cells received topographically correct synaptic inputs from appropriate cortical and subcortical regions, as assessed by performing monosynaptic rabies virus tracing. Overall, the authors showed that fetal neurons integrate adequately into the adult cerebral cortex, mature over time and re-establish a normal pattern of projection according to their original positional identity.

As previously discussed, in the last decade several groups worked to overcome the ethical and technical challenges of using fetal tissue, developing protocols to differentiate both mouse and human embryonic stem cells into neurons with appropriate cellular identity and area specification (Figures 1, 2).

Among the first, Pierre Vanderhaeghen and collaborators showed that murine PSC-derived pyramidal neurons grafted into the frontal cortex of neonatal mice and analyzed after 1 month displayed specific patterns of axonal projections corresponding to visual and limbic occipital cortex, despite the frontal site of transplantation (Gaspard et al., 2008). Furthermore, cultures transplanted at later stages of differentiation were enriched in later-born, superficial layer cell types. A similar pattern of projections was found 2 months after transplantation of human PSC-derived cortical neurons in frontal cortex of mouse pups, although 6 months post-transplantation the selectivity of these cells to contact the appropriate targets seems to decrease, with axonal projections reaching a wider range of regions, including motor and somatosensory areas (Espuny-Camacho et al., 2013). Noteworthy, Ideguchi and colleagues found no intrinsic regional identity in their PSC-derived pyramidal neurons, showing instead projection patterns coherent with the site of transplantation (Ideguchi et al., 2010). Thus, there may be a variability (likely depending on the protocol used for the differentiation) in the ability of the transplanted cells to retain the identity acquired *in vitro*.

In subsequent studies by Vanderhaeghen and collaborators, murine and human PSC-derived neurons were transplanted in a model of visual cortex lesion in adult mice and the re-establishment of the damaged pathways was assessed from 4 to 90 days after grafting (Michelsen et al., 2015). Grafted cells successfully differentiated, mainly in glutamatergic neurons, and showed reciprocal long-range axonal projections with targets of the damaged cortex such as the ipsilateral and contralateral cortex (layers II/III/IV), striatum (V), thalamus (VI) and midbrain/hindbrain nuclei (layer V). This study further confirmed that transplanted neurons display layer-specific patterns of projection in accordance with their molecular identity and consistent with the pattern of projection of native neurons of the visual cortex. Interestingly, these results were only obtained following homotopic transplantation in visual and not in motor cortex, as also confirmed by transplantation of fetal visual and motor cortical neural precursors. Human PSC-derived neurons maintained positional identity after transplantation and displayed the same projection properties of corresponding mouse cells, but required a longer time of maturation (Espuny-Camacho et al., 2018). Taken together, these results suggest that the success of transplantation in adult cortex is highly dependent on a strict match between the areal identity of the lesioned cortex and the positional identity of transplanted cells (Gaspard et al., 2008; Espuny-Camacho et al., 2013, 2018; Michelsen et al., 2015).

Among the first reports of mouse PSC used as cell therapy in animal models of stroke, Bühnemann et al. (2006) found that PSC-derived neural precursors transplanted into an Endothelin-1 (ET-1)-induced corticostriatal ischemic lesion, or in the perilesional striatum in rats, survived for up to 12 weeks and differentiated into distinct neuronal subtypes without expressing a specific cortical area-specific identity. However, the size of the surviving graft decreased from 4 to 12 weeks, probably due to graft rejection despite cyclosporine treatment, while paucity of fibers and high neuron mortality rate did not allow a proper analysis of the projection pattern.

Tornero et al., 2013 transplanted human IPSCs-derived, cortically committed or non-committed neural precursors, in the perilesional somatosensory cortex of a rat stroke model obtained by middle cerebral artery occlusion (MCAO). After 2 months they found that non-fated neural precursors cells (NPCs) proliferate more than their cortical counterpart. Both cell types migrated toward the ischemic lesion, but cortically fated cells differentiated more efficiently into TBR1- and SATB2-positive cortical neurons with a pyramidal morphology (Tornero et al., 2013). The same authors reported that grafted iPSC-derived cortical NPCs received synaptic inputs from the same areas as endogenous neurons (e.g., perilesional and contralateral cortex, ipsilateral and contralateral claustrum, specific thalamic nuclei, globus pallidus, etc.) as shown by the injection of a modified rabies virus that labeled monosynaptic afferents to grafted neurons. Finally, 6 months post-transplantation, immunoelectron microscopy confirmed that grafted neurons exhibited the ultrastructural features of mature neurons and formed synaptic contacts with host axon terminals, showing all of the ultrastructural criteria for synapses (Tornero et al., 2017).

Our group recently developed a protocol to produce either hippocampal or isocortical neurons that were then co-transplanted in healthy and lesioned brain regions. The analysis of *in vivo* projection pattern revealed that the different molecular identity acquired *in vitro* profoundly affect the different ability of these cells to extend axonal projections. In fact, we found that, accordingly to their molecular identity, isocortical neural precursors were not able to project once transplanted in the dentate gyrus (DG) of the hippocampus, contrary to PSC-derived hippocampal cells that properly elongated fibers to CA3 following endogenous hippocampal pathways. Instead, when grafted in their proper healthy cortical region, isocortical neurons sent projections toward the motor cortex and other cortical and subcortical targets. Interestingly, consistently with previous findings (de la Rosa-Prieto et al., 2017), fiber quantification revealed that the presence of an ischemic cortical lesion had an impressive impact in increasing the number of projections from isocortical cells, possibly through the same cellular mechanisms that spontaneously trigger and enhance post-stroke endogenous neural regeneration (Lindvall and Kokaia, 2015). Following stroke, the cortical cells elongated far reaching process that successfully integrated in motor pathways channeling in corticospinal tract segments, as the internal capsule (Terrigno et al., 2018b). However, PSC-derived isocortical cells transplanted into either intact or photothrombotic motor cortex failed in sending projections to thalamic nuclei and midbrain, in keeping with previous observations (Michelsen et al., 2015). This finding suggests that they might simply have maintained a general isocortical identity and that a further motor specification step may be required to generate also thalamic and midbrain projections.

Due to the clinical relevance and incidence of cortico-striatal lesions in humans, transplantation studies using neural precursors to rebuild lost circuitry focus mostly on sensory and motor cortices and striatum. However, since 1995 Fred Gage’s laboratory explored the possibility of transplanting cells in the hippocampus, publishing evidence that hippocampal neural stem cells long expanded *in vitro*, can integrate in the dentate gyrus and differentiate into mature neurons (Gage et al., 1995, 1998). Subsequently, it has been shown that pyramidal neurons obtained *in vitro*, integrate in the hippocampus, spreading throughout the CA3 region and the hilus, and elongate long projections to the contralateral hippocampus (Englund et al., 2002).

### Electrophysiological Properties of Grafted Neurons

Studying electrophysiological properties of grafted neurons is an unavoidable step to demonstrate the effective integration in the pre-existing circuitry and the formation of active synapses with the host tissue.

Different strategies can be adopted to study functional properties of grafted neurons, each one with its own limitations. Whole cell patch clamp in an *ex vivo* preparation permits the visualization of fluorescent grafted cells with the certainty to record from a single grafted neuron, allowing the experimenter to precisely study the input/output connectivity of the transplanted cells with surrounding neurons. However, with this *ex vivo* technique it is not possible to test the response of grafted neurons to more physiological environmental events, such as somatic or visual stimuli. On the other hand, during *in vivo* electrophysiological recordings it is challenging to unambiguously identify grafted cells within the cortex, posing uncertainty on the source of the recorded activity. However, appropriate strategies have been developed to overcome these limitations.

Several studies showed that neurons transplanted in the cortex differentiate and mature in functional neurons, which are characterized by appropriate electrophysiological properties of resting membrane potential, spontaneous and induced action potentials (APs) and spontaneous post-synaptic activity in voltage or current clamp configuration (Bühnemann et al., 2006; Daadi et al., 2009; Oki et al., 2012; Espuny-Camacho et al., 2013, 2018; Tornero et al., 2013; Michelsen et al., 2015). In one of the first reports, Bühnemann et al. (2006) studied the electrophysiological proprieties of murine neural progenitors transplanted in a stroke model. The authors showed appropriate voltage-gated sodium and potassium currents with reversal potential similar to control neurons and blocked them using specific drugs (TTX and TEA, respectively). In addition, they recorded APs and spontaneous excitatory post-synaptic currents in graft-derived cells indicating synaptic input. Moreover, spontaneous APs decreased their duration over time (3 to 6 weeks after transplantation) suggesting that maturation processes could take place in this window (Bühnemann et al., 2006).

Action potentials of murine and human grafted neurons, transplanted both in naïve and pathological conditions, have been recorded in spontaneous conditions or in voltage clamp configuration, using a depolarizing current (Daadi et al., 2009; Oki et al., 2012; Espuny-Camacho et al., 2013, 2018; Tornero et al., 2013; Michelsen et al., 2015). Interestingly, many of these studies reported both excitatory and inhibitory spontaneous post-synaptic activity of transplanted cells. This activity was totally blocked using AMPA, NMDA or GABA antagonists (CNQX, AP-V and Gabazine, respectively), indicating that transplanted cells successfully received inputs from resident neurons. Specific inputs from host neurons have been detected by electrically stimulating a cortical site far away from the transplant (e.g., the contralateral cortex) and visualizing post-synaptic activity (PSP or PSC) in some of the grafted cells, although action potentials were not reported, suggesting mature and functional host-to-neurons synapses but with a low density distribution (Oki et al., 2012; Espuny-Camacho et al., 2013, 2018; Tornero et al., 2013). In addition to electrical stimulation, optogenetic tools have been exploited to study afferent connectivity to grafted neurons. In fact, Tornero et al. (2013) reported host thalamic inputs to transplanted iPSC-derived cells that differentiated into pyramidal neurons and fast spiking interneurons. After light stimulation of the host neurons expressing Channelrhodopsin-2 in the ventral thalamic nuclei, EPSP and EPSC were detected in grafted neurons 3 months after transplantation in the ipsilesional somatosensory cortex (Tornero et al., 2017).

In another recent study, Wuttke et al. (2018) tested the callosal host inputs on grafted PSC-derived neurons, using a remarkably clean optogenetic approach. TdTomato-conjugated Channelrhodopsin-2 was *in utero* electroporated in correspondence to the layer II-III neuron production peak during corticogenesis (E14.5). At birth, they transplanted PSC-derived neurons in the contralateral homotopic region to the electroporaton site. Starting from P21, in acute slice, they patched grafted neurons (GFP+) surrounded by TdTomato fibers arising from contralateral callosal projecting neurons. In voltage-clamp configurations, grafted neurons showed spontaneous excitatory and inhibitory post-synaptic activity (EPSC and IPSC). After light stimulation of the contralateral ChR2-electroporeted neurons, authors found post-synaptic currents in grafted neurons, although no action potentials occurred (Wuttke et al., 2018). Notably, based on the latencies between the onsets of the light pulses and the light-evoked postsynaptic currents, the authors were able to distinguish between excitatory, monosynaptic transcallosal connections and polysynaptic, inhibitory inputs mediated by local interneurons. Finally, the authors assessed whether the grafted cells established functional contacts with the host neurons. Thus, after grafting ChR2-TdTomato cells in the neonatal cortex, they patched host neurons and optogenetically stimulated transplanted cells. They found that stimulation of grafted neurons induced EPSC in endogenous cells with a greater efficiency in ipsilateral compared to contralateral host neurons (Wuttke et al., 2018).

The main challenge of cell therapy in adult disease models is to obtain neurons behaving as the endogenous counterparts and properly responsive to environmental stimuli. In this regard, Pierre Vanderhaeghen and colleagues (Michelsen et al., 2015) tested the responsiveness to a visual stimulus of *in vitro* differentiated murine pyramidal visual cortex neurons, after their transplantation in the visual cortex of adult mice that was previously lesioned by ibotenic acid injection. The authors performed extracellular recordings in anesthetized animals 3–9 months after the transplant, placing the electrode in the center of the transplant and performing juxtacellular neurobiotin injection after the recordings. Grafted neurons showed spontaneous action potential firing with frequencies similar to control neurons. Then, they tested whether a physiological stimulus (1-s flash) was able to evoke spike activity in grafted neurons and found that half of recorded neurons increased their firing rate after flash presentation, with latencies and frequencies similar to naïve control neurons (Michelsen et al., 2015).

Falkner et al. (2016) reported functional maturation of murine grafted neurons in visual cortex after a selective degeneration of callosal projecting neurons in adult mice. The authors transplanted E14.5–E15.5 fetal cortical cells previously transduced with a calcium indicator. From 4 to 15 weeks post-transplantation they performed 2-photon imaging sessions, presenting gratings to the contralateral eye and reported a refinement over time of the orientation preference with sharp tuning properties in the later sessions (Falkner et al., 2016). These data are remarkable as the transplanted neurons appear to assume tuning properties indistinguishable from endogenous pyramidal cells.

Tornero et al. (2013) grafted human iPSCs-derived cortical neural precursor cells in the somatosensory cortex, 1 week after MCAO. Animals were subjected to extracellular recordings 3 months after transplantation, during tactile stimulation of different parts of the body. In these experiments the electrode was placed in the core of the transplant at least 70 μm far away from the border. Spike sorting allowed the identification of single unit responses. Authors reported an increased firing rate of some grafted neurons after stimulation of nose, forelimbs or hindlimbs, with latencies ranging from 9 to 32 ms. Moreover, spontaneous activity in some of the recorded neurons was inhibited by tactile stimulation but with a longer latency (46 to 85 ms) indicating a fine integration of these neurons in the cortical network, responding with an opposite directionality to stimuli from different parts of the body. In addition to single unit recordings, Local Field Potentials (LFPs) were also analyzed in this paper. Authors demonstrated that the evoked potential after nose stimulation was affected in untreated MCAO rats, showing a longer latency with respect to healthy animals. Grafting IPSC-derived cortical neurons rescued this deficit, shortening the latencies and making them similar to those of naïve rats (Tornero et al., 2017). Indeed, this is one of the few studies in which functional recovery after stroke was corroborated by electrophysiological evidences, suggesting that the therapeutic effect of the grafted neurons may be, at least in part, mediated by actual cellular replacement.

In animal models of epilepsy, several studies reported reduction of electrographic seizure activity mediated by neural transplantation (Hattiangady et al., 2008; Baraban et al., 2009; Maisano et al., 2012; Hunt et al., 2013; Southwell et al., 2014). In particular, it has been showed that grafting fetal interneurons, derived from human PSCs, in the hippocampus of a pilocarpine-induced temporal lobe epilepsy murine model, rescued the epileptic phenotype, with a very significant decrease in the number of seizures and amelioration of cognitive deficits and hyperactivity (Cunningham et al., 2014). 4 months post-transplantation, grafted interneurons were integrated in the hippocampus and showed spontaneous post-synaptic currents and regular membrane potential properties. In addition, efferent graft-to-host connections were tested using whole-cell patch clamp of endogenous neurons during optogenetic stimulation of Chr2-expressing cells. Post-synaptic activity induced by blue light stimulation in host neighboring neurons was totally abolished by the GABA antagonist bicuculline, thus demonstrating correct and mutual integration (Cunningham et al., 2014).

Overall, these results suggest that exogenous neurons can successfully integrate in a pre-existing neural network, developing mature synaptic contacts with resident neurons. In selected cases, transplanted neurons appear to contribute to rescue functional deficits. To determine the extent of integration within the host circuitry, it is now of paramount importance to assess how the grafted cells respond in awake animals during physiologically relevant behaviors. So far, only sensory stimuli have been used to drive grafted neurons. Thus, it would be of great interest to evaluate the responsiveness of transplanted cells during motor tasks. Hopefully, further research will shed light on these important questions.

### Therapeutic Effectiveness of Cell Replacement Therapies

The final goal of cell-based therapies is to improve recovery from functional deficits. In this context, beneficial effects of grafted cells can be ascribed to two, not mutually exclusive, mechanisms: (i) grafted cells could enhance functional recovery by secreting trophic factors and molecules with a neuroprotective/neuroplastic effect (also known as bystander effect); (ii) transplanted neurons could properly rebuild damaged circuitry, reestablishing lost sensorimotor synaptic connections. Consistently, cell-based therapy efforts are either directed toward grafting cells to produce and release trophic factors on site, or toward obtaining neurons with a specific identity in order to promote actual cell-replacement. Indeed, the fact that in several reports grafted cells were not detected in transplanted animals, despite an apparent functional improvement, indicates that: (i) the beneficial effect of the grafting was not related to long-term cell integration in the host neuronal circuitry and was exerted in the initial phase before the cells degenerated and (ii) the effect might be due to secretion of beneficial molecules released from grafted cells, or from resident cells stimulated by the grafting, in early post-implantation phase (Ramos-Cabrer et al., 2010; Oki et al., 2012; Hermanto, 2017).

Vascular endothelial growth factor (VEGF) has been implicated in mediating cell-induced functional recovery (Lee et al., 2007). In fact, neurons derived from human neural stem cells and overexpressing VEGF improved animal performance in the rotarod and limb placement tests when transplanted in the perilesional tissue after a hemorrhagic lesion, with early effects (8 days post-transplantation). Cells overexpressing VEGF also increased microvessels proliferation and diminished apoptotic cell death (Lee et al., 2007). Subsequently, Horie et al. (2011) confirmed that VEGF was responsible for the therapeutic effect of grafted neurons. They transplanted neurospheres in the peri-ischaemic cortex and observed an amelioration in the vibrissa-evoked forelimb placing test. Intriguingly, the recovery was totally abolished when VEGF was sequestered by a specific monoclonal antibody. In addition, histological investigations also reported a better blood brain barrier integrity, increased neovascularization and reduced inflammation (Horie et al., 2011). Moreover, the same authors showed that VEGF-mediated beneficial effects positively modulate axonal transport of host neurons, and stimulated axonal sprouting and dendritic plasticity (Andres et al., 2011).

It is generally accepted that grafted neurons could improve recovery by modulating neural plasticity. Indeed, transplanted interneurons induce plasticity in the host brain as reported by Southwell and colleagues, who demonstrated that grafting inhibitory interneurons in the adult visual cortex could re-open the critical period for ocular dominance plasticity (Southwell et al., 2010). In line with these results, hippocampal neural stem cells co-transplanted with astrocytes and microvascular endothelial cells in the hippocampus improved memory deficit after experimental stroke (Cai et al., 2015). Moreover, motor improvements have been reported following grafting of human derived neural precursors in rats with stroke (Daadi et al., 2008; Tornero et al., 2013; Tatarishvili et al., 2014), and after combination of grafting and exposure to enriched environment (Hicks et al., 2009). Recent findings confirmed the synergistic effect of coupling transplantation of murine E14.5 cortical NPCs with physical exercise, resulting in an increased and targeted fiber extension and a better motor improvement after aspiration of motor cortex in rats (Shimogawa et al., 2019).

## Conclusion

The final goal of neuroscientists working in cell therapy for brain diseases is to restore injured neural circuits, thus rescuing physiological impairments. Among the challenges faced by the clinical application of cell-based therapies is the choice of the cell source (fetal cells, embryonic PSCs or iPSCs). Indeed, both embryonic PSCs and fetal transplantations raise ethical issues, since fetuses are hardly a reliable and scalable source of material. Thus, iPSCs represent a good choice from a translational point of view, showing low immunogenicity and posing no ethical concerns. Human Leukocyte Antigen (HLA)-matched banking of iPSCs would reduce both the time and the cost associated with the derivation of clinically compliant induced pluripotent stem cell lines (reviewed in: Solomon et al., 2015) and several groups are making continuing efforts to improve the immune compatibility of PSCs, reducing the risk of immune rejection caused by HLA mismatching (Rong et al., 2014; Gornalusse et al., 2017; Xu et al., 2019). In addition, of great importance is the development of footprint-free reprogramming tools, to induce pluripotency while preserving the genetic stability of the reprogrammed cells and reducing the risk of tumorigenesis after transplantation, together with optimized protocols, allowing a more efficient cell replacement of the different damaged brain regions (reviewed in Martin, 2017). Finally, further preclinical studies should involve chemogenetic and optogenetic tools for stimulating activity of transplanted neurons and endogenous cells, during specific tasks in which these cells are involved. This would facilitate the strengthening of correct and effective synaptic contacts among grafted and naïve neurons, via mechanisms of Hebbian plasticity. Indeed, the establishment of new neuronal circuitry required for functional recovery after brain injuries likely depends on the same mechanisms of synaptic plasticity that drive experience-dependent refinement of connections during brain development.

## Author Contributions

All authors revised, wrote, and discussed the manuscript.

## Conflict of Interest Statement

The authors declare that the research was conducted in the absence of any commercial or financial relationships that could be construed as a potential conflict of interest.
